# The Gaze-Cueing Effect in the United States and Japan: Influence of Cultural Differences in Cognitive Strategies on Control of Attention

**DOI:** 10.3389/fpsyg.2017.02343

**Published:** 2018-01-15

**Authors:** Saki Takao, Yusuke Yamani, Atsunori Ariga

**Affiliations:** ^1^Faculty of Science and Engineering, Waseda University, Tokyo, Japan; ^2^Department of Psychology, Old Dominion University, Norfolk, VA, United States; ^3^Department of Behavioral Science, Hiroshima University, Hiroshima, Japan

**Keywords:** gaze-cueing effect, cultural differences, cued attention, Asians, Westerners, cognitive strategies

## Abstract

The direction of gaze automatically and exogenously guides visual spatial attention, a phenomenon termed as the *gaze-cueing effect*. Although this effect arises when the duration of stimulus onset asynchrony (SOA) between a non-predictive gaze cue and the target is relatively long, no empirical research has examined the factors underlying this extended cueing effect. Two experiments compared the gaze-cueing effect at longer SOAs (700 ms) in Japanese and American participants. Cross-cultural studies on cognition suggest that Westerners tend to use a context-independent analytical strategy to process visual environments, whereas Asians use a context-dependent holistic approach. We hypothesized that Japanese participants would not demonstrate the gaze-cueing effect at longer SOAs because they are more sensitive to contextual information, such as the knowledge that the direction of a gaze is not predictive. Furthermore, we hypothesized that American participants would demonstrate the gaze-cueing effect at the long SOAs because they tend to follow gaze direction whether it is predictive or not. In Experiment 1, American participants demonstrated the gaze-cueing effect at the long SOA, indicating that their attention was driven by the central non-predictive gaze direction regardless of the SOAs. In Experiment 2, Japanese participants demonstrated no gaze-cueing effect at the long SOA, suggesting that the Japanese participants exercised voluntary control of their attention, which inhibited the gaze-cueing effect with the long SOA. Our findings suggest that the control of visual spatial attention elicited by social stimuli systematically differs between American and Japanese individuals.

## Introduction

A typical scene contains an array of visual objects, yet human observers, limited-capacity information processors, cannot process all of the objects simultaneously. One function of visual attention is to select objects of behavioral interest and ignore the others. Attentional selection can operate spatially (e.g., Posner, 1980; Treisman and Gelade, 1980; Posner and Cohen, 1984; Wolfe, 1994; Spence and Driver, 1997; Itti and Koch, 2000) based on an observer’s prior knowledge of the characteristics or location of a target. However, such top-down, or endogenous, deployment of attention can be involuntarily modulated by exogenous visual cues.

Posner et al. (1980) investigated how bottom-up cueing modulates the allocation of spatial attention. Participants were instructed to detect a target presented peripherally in a left or right rectangular placeholder while fixating on the center of the display. When the duration of stimulus onset asynchrony (SOA) was short (100 ms), participants’ reaction times (RTs) were markedly shorter when a flash of the peripheral placeholder served as a valid cue for the location of the target than when it was an invalid cue. Critically, the location of the flash (cue) did not predict the upcoming target location (cue validity = 50%), suggesting that the *cueing effect* arises involuntarily with a relatively short SOA. However, this cueing effect was eliminated (or reversed due to inhibition of return, Klein, 2000) when the cue-target SOA duration was long (more than 300 ms), presumably allowing observers time to reorient attention to the target following the onset of the peripheral cue. These findings suggest that the salient visual event exogenously and involuntarily oriented attention before the visual system could endogenously direct attention to the target.

Furthermore, such exogenous modulation of visual attention can occur with bottom-up cues that are not physically salient but socially important (e.g., Friesen and Kingstone, 1998; Driver et al., 1999; Langton and Bruce, 1999, 2000; Kingstone et al., 2000; Langton et al., 2000; Ariga and Watanabe, 2009). For example, Driver et al. (1999) investigated whether a social sign could exogenously modulate an observer’s visual attention similar to the non-predictive salient cue used by Posner et al. (1980). They manipulated the relationship between the non-predictive gaze direction of the central face (i.e., left-directed or right-directed eye gaze cue) and the target location (i.e., left or right of the central face). The time to discriminate the target was shorter when the target appeared in the direction of the seen gaze as compared to the opposing direction, a phenomenon called the *gaze-cueing effect*. Because the gaze-cueing effect emerged at the short SOA (100 ms), these findings suggest that social signs (i.e., gaze direction) are automatically detected and involuntarily modulate the deployment of attention.

However, the gaze-cueing effect has been observed at a relatively long SOA (700 ms), the time interval when a voluntary shift of visual attention occurs (Posner, 1980; Spence and Driver, 1997), whereas typically, the peripheral cueing effect is eliminated (or reversed) with a long SOA (e.g., Posner et al., 1980; Posner and Cohen, 1984; Spence and Driver, 1997). In general, if the preceding non-predictive cue attracted attention exogenously, the visual system could voluntarily reorient attention during a long SOA duration for the upcoming target; however, this was not the case for the gaze-cueing effect. Driver et al. (1999) interpreted this discrepancy as a result of the extended time course of the automatic attentional orienting, particularly in response to the gaze cue. However, the mechanisms underlying the extended time course of attentional orientation remain unclear.

Cultural differences in cognitive processing strategies may influence the magnitude of the gaze-cueing effect at relatively long SOAs. Previous work suggests that Westerners tend to process objects in the environment analytically, independent of context, whereas Asians tend to engage in context-dependent holistic processing (e.g., Kitayama et al., 2003; Nisbett and Miyamoto, 2005). For example, Masuda and Nisbett (2001) found that when viewing a video clip of an underwater scene, American and Japanese observers noticed different aspects of the scene. The Americans predominantly reported salient focal objects (e.g., moving fish), while Japanese observers often commented on contextual objects (e.g., background seaweed). Furthermore, previous findings suggest that non-visual context (e.g., vocal tone; Ishii et al., 2003, or social situations involving a person’s behavior; Miller, 1984; Morris and Peng, 1994) as well as visual context (e.g., object information surrounding a focal object or background information; Ji et al., 2000; Masuda and Nisbett, 2001; Nisbett and Miyamoto, 2005) can influence attentional orientation. Thus, previous research supports the view that Westerners tend to use analytic processes to perform a cognitive task, whereas Asians tend to approach the task more holistically when controlling attention in a top-down manner.

In the current context of the gaze-cueing effect, we hypothesized that, among Western participants, the gaze-cueing effect would persist with a long SOA. Western participant’s visual system, a more analytical processor, is less susceptible to contextual information that requires a holistic interpretation of the relationship between the gaze direction and the subsequent target location in our study. Essentially the information of the direction of the seen gaze is not predictive of the target location. This strategy makes the visual system vulnerable to exogenous social signs and less likely to redirect attention with a long SOA. Consistent with this hypothesis, a number of previous studies in Western countries have demonstrated the gaze-cueing effect with relatively long cue-target SOAs (e.g., Friesen and Kingstone, 1998; Driver et al., 1999; Mansfield et al., 2003; Frischen and Tipper, 2006; Bayliss et al., 2007). That is, Western participants demonstrated the gaze-cueing effect at short and long SOAs despite the fact that the direction of gaze was not predictive because their processing strategy emphasized the importance of gaze cues over the knowledge that the cues were not predictive. Conversely, although Asian participants demonstrated the gaze-cueing effect at longer SOAs, the magnitude was smaller than that typically observed in Western participants (Tokunaga and Miyatani, 2008; Kawai, 2011; Nishiyama and Kawaguchi, 2011, 2012; but see Akiyama et al., 2006). For example, Kawai (2011) graphically showed a robust gaze-cueing effect at shorter SOAs (105–300 ms), but not longer SOAs (600–1005 ms), with Japanese participants, although the interaction was not significant. A similar effect was observed with Japanese children in a developmental study (Senju et al., 2004). We hypothesized that, because Asians tend to be holistic processors, they would be less susceptible to non-predictive gaze cues, thus allowing more efficient re-deployment of attention at longer SOAs than would Westerners. We replicated the gaze-cueing effect at a short SOA and then investigated whether the gaze-cueing effect emerged differently in American and Japanese participants, in identical experiments conducted in the United States and Japan with a long SOA. In fact, the literature on spatial cueing suggests that top-down guidance of attention cannot override bottom-up, data-driven guidance within the short SOA (Muller and Rabbitt, 1989; Remington et al., 1992). Therefore, the gaze-cueing effect was expected to occur for the short SOA irrespective of culture as consistently reported by previous research with Japanese participants (Senju et al., 2004; Akiyama et al., 2006; Tokunaga and Miyatani, 2008; Kawai, 2011; Nishiyama and Kawaguchi, 2011, 2012). More importantly, we predicted that (1) American participants would demonstrate the gaze-cueing effect at the long SOA and (2) Asian participants would not demonstrate a comparable gaze-cueing effect at the long SOA.

## Experiment 1: The Gaze-Cueing Effect in American Participants

### Methods

#### Ethics Statement

Experiment 1 was reviewed and approved by the Institutional Review Board of Old Dominion University, Norfolk, VA, United States. Written informed consent was obtained from all participants prior to the experiment.

#### Participants

We recruited 30 American participants (4 males, mean age = 19.37 years, *SD* = 1.43 years) from the Old Dominion University student community. All participants reported normal or corrected-to-normal visual acuity. Participants were blinded to the purpose of the study and received course credit for participation.

#### Stimuli

The stimuli were similar to those of Friesen and Kingstone (1998): a black line drawing of a round face subtending 6.8° containing two eyes (two open circles subtending 1.0°), a nose (one circle subtending 0.2°), and a mouth (one straight line 2.2° in length) drawn on a white background. The eyes were separated by a 2.0° space and were located 0.8° above the central horizontal axis. The nose was located at the center of the display, and the mouth was located 1.3° below the nose. Black pupils (filled-in circles subtending 0.5°) served as cues and were just touching the left or just touching the right in the eyes. A black dot subtending 0.6° was used as the target and was placed 5.9° to the left or right of the fixation point (a red dot).

#### Apparatus

Stimuli were presented on a Samsung T24C550 23.6″ LED monitor with 1980 × 1080 resolution. The experiment was controlled by a Dell Optiplex 9020 running MATLAB with the Psychtoolbox extension (Brainard, 1997; Pelli, 1997). Participants viewed the monitor from a distance of approximately 57 cm. The experiments were conducted in a quiet room with dimmed light.

#### Procedure

**FIGURE 1:**
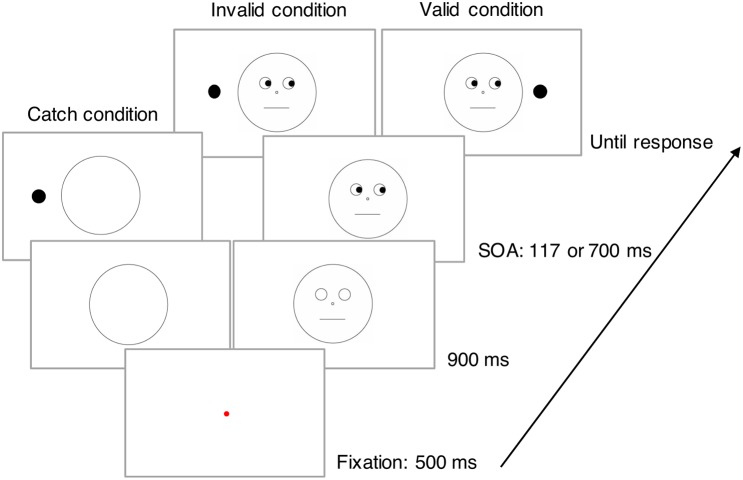
Schematic illustration of each condition in Experiment 1.

After participants pressed the keyboard space key, a fixation point appeared at the center of the display for 500 ms (**Figure 1**). Then, a face with blank eyes appeared for 900 ms after which black pupils appeared in the eyes indicating the gaze direction as left or right. A target dot then appeared to the left or right side of the face with an SOA of 117 ms (short) or 700 ms (long). The participants’ task was to indicate the location of the dot as quickly and accurately as possible. Participants were instructed to press the left arrow key on the keyboard with their left index finger when the target dot appeared on the left side of the face. They were asked to press the right arrow key with their right index finger when the dot appeared on the right side of the face. Given that several previous studies have investigated covert attention in the gaze-cueing paradigm (e.g., Friesen and Kingstone, 1998; Driver et al., 1999; Akiyama et al., 2006), we did not monitor eye movements in this study.

The target appeared at the location where the gaze was directed (valid condition) in one-third of the trials and at the location where the gaze was not directed in one-third of the trials (invalid condition). The remaining trials were catch trials in which only the face outline (an open circle) appeared, and participants were required to withhold their responses (Takahashi and Watanabe, 2013). This condition was used to prevent response preparation before target onset and to encourage participants to attend to the eyes. Participants were informed in advance that the gaze direction did not predict the target location (validity = 50%). The experiment included 384 experimental trials (128 trials each under the valid, invalid, and catch conditions).

### Results and Discussion

**FIGURE 2:**
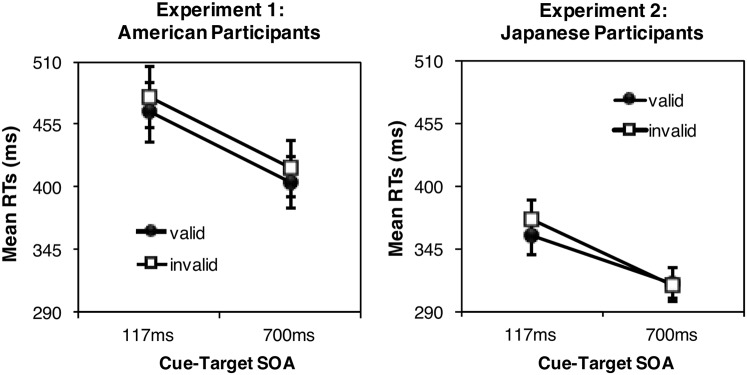
Mean reaction times (RTs) under each condition as a function of stimulus onset asynchrony (SOA) in Experiment 1 **(Left)** and Experiment 2 **(Right)**. Error bars indicate between-participants 95% confidence intervals.

The median RT for correct localization of the target under each condition was calculated for each participant and analyzed using a 2 (Condition: valid vs. invalid) × 2 (SOA: short vs. long) repeated-measures analysis of variance (ANOVA). **Figure 2** (Left) shows the mean RTs for the experimental conditions (short SOA × valid vs. invalid: 465.85 ms vs. 478.92 ms; long SOA × valid vs. invalid: 403.72 ms vs. 416.03 ms). RTs were shorter under the valid condition than under the invalid condition [*F*(1,29) = 11.95, *p* < 0.01, $\eta_{p}^{2}$ = 0.29], demonstrating the gaze-cueing effect. Furthermore, RTs were faster under the long SOA than under the short SOA condition [*F*(1,29) = 232.05, *p* < 0.01, $\eta_{p}^{2}$ = 0.89]. However, no significant interaction effect was found [*F* < 1, *n.s.*] indicating that the magnitude of the gaze-cueing effect was similar under both SOA conditions (13.07 ms vs. 12.31 ms, for the short and long SOA conditions, respectively). The error rates, proportion of localization errors, were low under each condition (0.23% on average) and not significantly different between conditions [*F* < 1, *n.s.*] indicating no speed-accuracy trade-off. The error response (false alarm) rate on the catch trials was low (5.52% on average) and did not significantly differ between conditions [*F* < 1, *n.s.*; **Table 1**] indicating that the catch trials were effective. In a further analysis, we divided the data into male (*N* = 4) and female (*N* = 26) groups to investigate the gender effect. The small number of males did not allow for statistical comparison; however, the trends in male and female responses did not differ graphically.

**Table 1:** Percent error rate (standard error) for each condition in Experiment 1.

	SOA-117ms	SOA-700ms
Valid condition	0.16 (0.09)	0.26 (0.11)
Invalid condition	0.26 (0.13)	0.21 (0.12)
Catch condition	5.83 (1.19)	5.21 (1.10)


The gaze-cueing effect was observed under both SOA conditions. This pattern is consistent with previous findings (e.g., Driver et al., 1999). Although participants were informed that the gaze direction did not predict the location of the target, they did not suppress the gaze-driven attentional orienting under the long SOA condition. We interpreted this finding as the result of the extended time course of attentional orienting given the cognitive strategy used by the Americans (e.g., Nisbett and Miyamoto, 2005). Because Westerners tend to focus on a salient event (the gaze direction) independently of its context (the cue validity), it is likely that American participants processed the gaze cue analytically, thus eliciting the gaze-cueing effect. In Experiment 2, we investigated whether the Japanese participants would exhibit the gaze-cueing effect at the short, but not long SOA. Japanese individuals tend to focus more on the context in which an object is presented (i.e., the unpredictability of the gaze direction) than do Americans.

## Experiment 2: The Gaze-Cueing Effect With Japanese

### Methods

#### Ethics Statement

Ethics approval was not required by Rissho University guidelines or Japanese national regulations. The experiment was approved by the Department of Psychology at Rissho University. Written informed consent was obtained from all participants prior to the experiment.

#### Participants

We recruited 30 Japanese participants (14 males, mean age = 20.30 years, *SD* = 1.27 years) from Rissho University, Tokyo, Japan. All participants reported normal or corrected-to-normal visual acuity. Participants were blinded to the purpose of the study and volunteered for the experiment.

#### Apparatus

Stimuli were presented on a Dell P2414HB 24″ Full HD monitor with 1980 × 1080 resolution. The experiment was controlled by a Dell Precision T3610, running MATLAB with the Psychtoolbox extension (Brainard, 1997; Pelli, 1997). Participants viewed the monitor from a distance of approximately 57 cm. The experiments were conducted in a quiet room, with dimmed light.

#### Stimuli and Procedure

The stimuli and procedure were identical to those used in Experiment 1.

### Results and Discussion

Data analyses were identical to those performed in Experiment 1. **Figure 2** (Right) shows the mean RTs averaged across participants (short SOA × valid vs. invalid: 357.12 ms vs. 370.72 ms; long SOA × valid vs. invalid: 315.08 ms vs. 313.55 ms). Similar to the results in Experiment 1, the RTs under the valid condition were shorter than those under the invalid condition [*F*(1,29) = 8.69, *p* < 0.01, *$\eta_{p}^{2}$* = 0.23], and the RTs were faster under the long SOA than under the short SOA condition [*F*(1,29) = 160.46, *p* < 0.01, *$\eta_{p}^{2}$* = 0.85]. However, the interaction effect was significant [*F*(1,29) = 23.17, *p* < 0.01, *$\eta_{p}^{2}$* = 0.44], indicating that the gaze-cueing effect was observed for the short SOA [*M* = 357.12 ms vs. 370.72 ms for the valid and invalid conditions, respectively; *paired-samples t*(29) = 5.91, *p* < 0.01, *d* = 1.08], but not the long SOA [*M* = 315.08 ms vs. 313.55 ms for the valid and invalid conditions, respectively; *paired-samples t*(29) = 0.59, *p* = 0.54, *d* = 0.09]. The error rates and proportion of localization errors were low under each condition (0.26% on average) and were not significantly different between conditions [*F* < 1, *n.s.*] indicating no speed-accuracy trade-off. The error response (false alarm) rate on the catch trials was low (3.36% on average) and was not significantly different between conditions [*F* < 1, *n.s.*] (**Table 2**) indicating that the catch trials were effective. As in Experiment 1, male (*N* = 14) and female (*N* = 16) data were analyzed to investigate the gender effect. We subtracted the median RT for the valid condition from that of the invalid condition for each participant to provide an index of the gaze-cueing advantage. This was assessed using a two-way ANOVA with SOA and gender as factors. We found no significant main effect of gender [*F*(1,28) = 0.24, *p* > 0.05, $\eta_{p}^{2}$ = 0.01]; however, the main effect of SOA was significant [*F*(1,28) = 22.15, *p* < 0.01, $\eta_{p}^{2}$ = 0.44]. The interaction between gender and SOA was not significant [*F*(1,28) = 0.18, *p* > 0.05, $\eta_{p}^{2}$ = 0.01].

**Table 2:** Percent error rate (standard error) for each condition in Experiment 2.

	SOA-117ms	SOA-700ms
Valid condition	0.26 (0.11)	0.16 (0.09)
Invalid condition	0.21 (0.12)	0.42 (0.16)
Catch condition	4.48 (0.72)	2.24 (0.52)


## Cross-Experiment Analysis

We performed a cross-experiment analysis to compare the magnitude of the gaze-cueing effect at the long SOA between the American and Japanese participants. The gaze-cueing advantage of the long SOA was significantly larger in the American participants [*independent-samples t*(58) = 2.15, *p* < 0.05, *d* = 0.03]; however, we found no difference between groups at the short SOA [*independent-samples t*(58) = -0.10, *p* > 0.05, *d* = 0.56].

It is possible that the absence of the extended cueing effect in Japanese participants was the result of a floor effect, because their RTs were generally shorter than those of the American participants. To rule out this possibility, we examined the correlations between the magnitude of the gaze-cueing effect and the median RTs of individuals for each SOA. No significant correlations were found [Experiment 1: 117-ms SOA, *r* = 0.12, *p* = 0.52; and 700-ms SOA, *r* = 0.12, *p* = 0.53; Experiment 2: 117-ms SOA, *r* = -0.03, *p* = 0.87; and 700-ms SOA, *r* = 0.14, *p* = 0.45], suggesting that the gaze-cueing effect occurred independently of RT. This finding is supported by that of Friesen and Kingstone (1998) who reported a mean RT of 350 ms at SOAs between 100 and 700 ms on a localization task. This is comparable to the values of the Japanese participants in our study. Friesen and Kingstone (1998) found a significant gaze-cueing effect at the 100-ms and 700-ms SOAs, suggesting that the elimination of the extended cueing effect among the Japanese participants in Experiment 2 was not due to a floor effect.

As expected, the short, but not the long SOA, elicited a reliable gaze-cue effect in Japanese participants. This occurred despite the fact that the stimuli, procedure, and task were identical to those used for the American participants in Experiment 1. Furthermore, the cross-experiment analysis revealed that cultural factors had a measurable and statistically significant effect on the gaze-cueing advantage at the long SOA. Our findings support the hypothesis that Asians intentionally inhibited gaze-driven attentional orienting based on the *a priori* knowledge that the gaze direction did not predict the target location.

## General Discussion

We replicated the gaze-cueing effect with a short SOA for Western and Asian participants and then compared the effect of a longer SOA on the gaze-cueing effect. We hypothesized that at the longer cue-target SOA the magnitude of the gaze-cueing effect would be influenced by the differing cognitive strategies typical of Western and Asian individuals (i.e., analytic vs. holistic processing, respectively; Kitayama et al., 2003; Nisbett and Miyamoto, 2005). In Experiment 1, American participants demonstrated the gaze-cueing effect under the short and long SOA conditions, indicating that they were influenced by the salient gaze direction despite the SOA duration. This finding suggests that American participants did not rely on the predictive gaze direction information to orient their attention. Conversely, in Experiment 2, the Japanese participants demonstrated the gaze-cueing effect at the short, but not the long SOA, indicating that they voluntarily controlled their attention in response to the contextual information that the gaze cue was non-predictive. These findings support our hypothesis that an extended time course of the attentional orienting (Driver et al., 1999) reflects the cognitive processing strategies of Westerners.

Bonduroglu et al. (2009) used a change detection task to compare visual information processing in Asians and Westerners. They found that East Asians were better at detecting changes at the visual periphery, whereas Westerners were better at detecting changes in the center of a visual field. Therefore, because we manipulated the gaze direction in the center of the display, American participants may have been more responsive to the central cueing gaze than Japanese participants. This may explain the cultural differences in the gaze-cueing effect. Furthermore, Markus and Kitayama (1991) proposed that views of the self differed between Western and East Asian cultures, such that Westerners tend to view the self independently from others, whereas East Asians tend to view the self in the context of others, valuing the importance of harmonious interdependence with others. This culture-based view of the self may have formed the basis of the participants’ cognitive strategy in our gaze-cueing task: the Japanese were attentive to the reliability of others’ gaze within the context of the task and preempted the gaze-cueing effect at the long SOA. Conversely, Americans did not focus on the reliability of the other and demonstrated the gaze-cueing effect despite the context. These cultural differences may underlie the fact that the extended cueing effect was elicited by social signs (gaze cues) in our study, but not by non-social signs in previous studies (traditional flash cues, e.g., Posner et al., 1980; Posner and Cohen, 1984; Spence and Driver, 1997).

Furthermore, we investigated whether the gaze-cueing effect, at the relatively long SOA, demonstrated by American participants (Experiment 1) was the result of race or cultural factors. The participants in Experiment 1 included 14 African-Americans, 11 Caucasians, and the remaining participants were of various races (e.g., Hispanic). We reanalyzed the data according to the two main races (African-American and Caucasian). For African-Americans, the main effects of condition and SOA were significant (*p* < 0.01); however, the interaction was not significant. For Caucasians, the main effect of condition was marginally significant (*p* = 0.07), the main effect of SOA was significant (*p* < 0.01), and the interaction was not significant. The results were not statistically different, suggesting that cultural factors, not race, were responsible for the gaze-cueing effect at the long SOA.

## Conclusion

Our findings offer novel insights into the way in which culture-specific cognitive strategies can influence the dynamic control of visual attention in space. Specifically, we demonstrated that the gaze-cueing effect at a relatively long SOA systematically varies between American and Japanese participants: the magnitude of the gaze-cueing effect at the longer SOAs persisted in the Americans but diminished in the Japanese participants. Our findings generally support the existence of cultural differences in cognitive processing strategies (Kitayama et al., 2003; Nisbett and Miyamoto, 2005). That said, other potential factors should be considered, such as differences in cultural norms pertaining to eye contact, eye shape, and general trust. First, American culture generally values eye contact in social situations, whereas Japanese culture does not. Therefore, differences in the perception of eye contact by others may have played a role in determining the magnitude of the gaze-cueing effect in the American and Japanese participants. Second, differences in typical eye shapes may have affected the results. We used physically identical faces in the American and Japanese experiments so we could directly compare the gaze-cueing effect while minimizing potential effects of race, gender, and individual preferences. Third, some studies indicate that Asians tend to modulate their eye movement patterns in response to context information more sensitively than Westerners (e.g., Chua et al., 2005; Boland et al., 2008; but see also Rayner et al., 2007, 2009; Evans et al., 2009). Such differences in eye movement patterns between different culture systems might have contributed to the absence of gaze-cueing effect among Japanese participants at the long SOA; even if that was the case, it would be at least cultural differences in visual processing strategies that determined the presence/absence of the gaze-cueing effect at the long SOA. Fourth, Yamagishi and Yamagishi (1994) found that American participants reported higher levels of trust in others than Japanese participants. Our findings may reflect individual differences in general trust levels. Lastly, it is possible that faster responses of the Japanese participants than the American participants in general may be attributed to extraneous factors such as differences in testing environments (e.g., apparatus) in different countries. Future research should cross culture (Japanese vs. American) and testing locations (Japan and the United States) to assess whether the testing environment is responsible for the current result, independent of the effect of culture systems. Further investigations are necessary to understand more fully the mechanisms underlying the gaze-cueing effect.

## Author Note

Thanks are due to Mariska Kret, two reviewers and Molly Liechty for helpful comments on an earlier draft of the manuscript.

## Author Contributions

ST, YY and AA designed the work, collected, analyzed and interpreted the data, drafted and approved the manuscript.

## Conflict of Interest Statement

The authors declare that the research was conducted in the absence of any commercial or financial relationships that could be construed as a potential conflict of interest.
